# Algae-dominated metaproteomes uncover cellular adaptations to life on the Greenland Ice Sheet

**DOI:** 10.1038/s41522-025-00770-2

**Published:** 2025-09-09

**Authors:** Helen K. Feord, Anke Trautwein-Schult, Christoph Keuschnig, Anne Ostrzinski, Elisa K. Peter, Carsten Jaeger, Jan Lisec, Rey Mourot, Ravi Sven Peters, Ozan Çiftçi, Martyn Tranter, Alexandre M. Anesio, Dörte Becher, Liane G. Benning

**Affiliations:** 1 GFZ Helmholtz Centre for Geosciences, Potsdam, Germany; 2 Institute of Microbiology, University of Greifswald, Greifswald, Germany; 3 Department of Earth Sciences, Freie Universität Berlin, Berlin, Germany; 4 Bundesanstalt für Materialforschung und -prüfung, Berlin, Germany; 5 Aix Marseille Université, Université de Toulon, CNRS, IRD, MIO, Marseille, France; 6 Department of Environmental Science, Aarhus University, Roskilde, Denmark

**Keywords:** Environmental microbiology, Water microbiology, Cellular microbiology

## Abstract

Eukaryotic algae-dominated microbiomes thrive on the Greenland Ice Sheet (GrIS) in harsh environmental conditions, including low temperatures, high light, and low nutrient availability. Chlorophyte algae bloom on snow, while streptophyte algae dominate bare ice surfaces. Empirical data about the cellular mechanisms responsible for their survival in these extreme conditions are scarce. This knowledge gap was addressed by quantifying proteins for both algal taxa from samples on the southern margin of the GrIS. We show that the streptophyte glacier ice algae have a relative enrichment in proteins involved in environmental signaling and nutrient transport, indicative of cellular readiness to dynamically respond to extreme GriS environmental cues, linked, for example, to photoprotection and the rapid update of scarce nutrients. In contrast, the chlorophyte snow algae have a high abundance of proteins linked to lipid and nitrogen metabolisms, providing evidence for the biological processes sustaining the cellular carbon and nitrogen stores necessary for survival in an oligotrophic environment. We also identify proteins in both taxa linked to the synthesis and breakdown of key cellular pigments. Our study gives novel insights into the cellular biology of these algae and their adaptation to extreme environments.

## Introduction

Eukaryotic algae and their associated microbiomes live on terrestrial snow and ice substrates globally. Such habitats are generally oligotrophic, undergo regular freeze-thaw cycles, and often receive high photosynthetically active and UV radiation^1–3^, creating a unique and extreme set of living conditions. Chlorophyte algae, including the *Sanguina* and *Chloromonas* genera (Chlorophyceae), live on snow, often in carotenoid-rich hypnozygote stages^3,4^. The two streptophyte *Ancylonema* spp., *A. alaskanum and A. nordenskioeldii* (Zygnematophyceae)^5^, dominate ice surface microbiomes, mostly in a vegetative stage with their characteristic intracellular purple/brown pigment purpurogallin^6^. Both the snow algae and glacier ice algae act as primary producers in the ecosystem in which they live^7,8^, along with heterotrophs such as fungi^9,10^ and bacteria^10^, as well as viruses^11^. Both algal taxa enhance snow and ice melt via surface darkening^12,13^. Functional information about how these eukaryotes survive and grow on snow and ice is essential for predicting how they will respond to future climate change, which will lengthen melt seasons. Data is available regarding the biogeography^14,15^, ecology^15,16^, and photophysiology^17,18^, as well as pigment and metabolomic profiles^17–19^, of both snow and glacier ice algae. Functional data from genome^20,21^ and metagenome sequencing^22^ is also beginning to be explored. However, currently we have no information regarding protein profiles of either of these taxa from environmentally relevant conditions, as we lack any metaproteome datasets.

Identification and quantification of these algae’s proteins help our understanding of their cellular structure, function, and signaling system^23,24^, and such information is fundamental for assessing their adaptation to the harsh conditions they thrive in. Furthermore, the ubiquitous decoupling of transcript and protein levels in many species^25^ makes protein quantification necessary to functionally characterize cellular phenotypes. Given the difficulty of accurately recreating the unique abiotic conditions of supraglacial environments in laboratory settings, the quantification of proteins directly from environmental samples, using a metaproteomic approach, is critical to understand the in situ functioning of these algae. Furthermore, metaproteomics is especially useful for taxa behaving differently in culture compared to their natural environment, which is the case for cellular morphology and pigment production for many snow algae^26^ and all glacier ice algae^27,28^. Metaproteomic approaches have helped quantify marine eukaryotic algal proteins^29,30^, but so far, no such data is available for algae-dominated terrestrial snow and ice samples.

Here, we address this important knowledge gap by providing a first quantification of the protein profiles of blooming chlorophyte snow and streptophyte glacier ice algae from the Greenland Ice Sheet (GrIS). We characterize a streptophyte proteome enriched in proteins involved in environmental signaling, the shikimate pathway, and nutrient transport. On the contrary, the chlorophyte metaproteome is enriched in functions involved in lipid metabolism, carotenoid synthesis and breakdown, and nitrogen metabolism.

## Results

### Metaproteome workflow and taxonomic assignment of protein groups

**Fig. 1:**
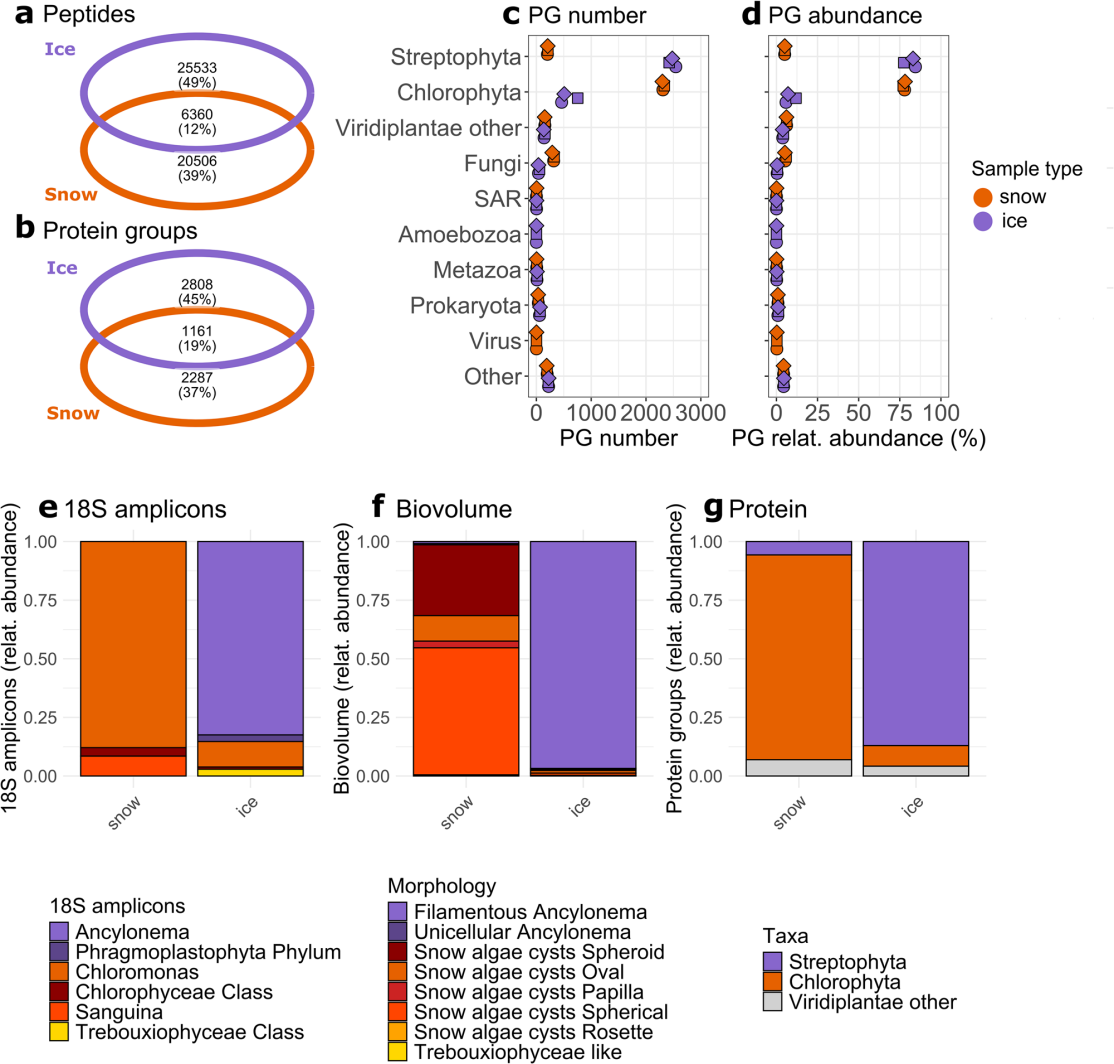
Protein group quantity and taxonomy in the snow and ice samples. Total number and percentage of quantified **a** peptides and **b** protein groups (PGs), and overlap between snow and ice samples. Taxonomy of PGs by **c** number of quantified PGs, and **d** relative sum weighted spectra, with each point representing a replicate (shown with different symbols). Mean relative abundance of algal taxa in snow and ice samples based on **e** 18S amplicon sequencing, **f** biovolume (calculated based on cell counts and imaging; for morphologies matching the descriptions see Supplementary Fig. 3), and **g** sum weighted spectra of algal taxa based on PG quantity. PG protein group, relat. abundance relative abundance, SAR Stramenopiles-Alveolates-Rhizarians.

Proteins from red snow and dark ice samples collected in summer 2021 from the southern margin of the GrIS (Supplementary Fig. 1a–e; “Methods”) were extracted in triplicate and analyzed with liquid chromatography with tandem mass spectrometry (LC-MS/MS). MS spectra were analyzed using a predicted protein database compiled from GrIS metatranscriptomes from samples collected during separate field campaigns^11^. Our metaproteomics workflow was successful for both sample types, with a total of 7517 protein groups (PGs) identified, and 6256 PGs quantified (identified in at least two replicates). The snow and ice samples showed minimal overlap in shared peptides (12%, Fig. 1a) and quantified PGs (19%, Fig. 1b), indicating a strong distinction in their peptide and protein identities. In addition, the large and relatively comparable number of peptides and PGs identified in both sample types confirmed that the database used catered well for both sample types (Fig. 1a, b).

Taxonomic assignment of PGs revealed that the majority of the PGs (>2500) across all samples belonged to Viridiplantae in both sample types (Fig. 1c, d). Snow samples were predominantly composed of chlorophyte PGs (>2200 PGs, ~77% total abundance), while ice samples were dominated by streptophyte PGs (>2300 PGs, ~80% total abundance). In both sample types, ~150 of the Viridiplantae PGs were not assigned to a specific phylum (“Viridiplantae other”, Fig. 1c, d). Based on 18S amplicon sequencing data, algal taxa in the snow samples were dominated by chlorophyte species, primarily from the *Chloromonas* genus, with minor contributions from *Sanguina* spp. (Fig. 1e, Supplementary Fig. 2a, b). In contrast, ice samples were dominated by the streptophyte *Ancylonema* spp. (~80%; Fig. 1e, Supplementary Fig. 2a, b). This finding was corroborated by imaging, cell counts, and biovolume quantification, allowing us to clearly distinguish and identify multiple cell types for the chlorophyte snow algae (indicating different life stages of the same or different species) and unicellular and filamentous *Ancylonema* spp. (Fig. 1f, Supplementary Fig. 3). The relative chlorophyte vs. streptophyte abundance from our 18S amplicon and biovolume datasets mirrors the trends found for protein in both sample types (Fig. 1g). Fungal PGs were also abundant in snow (~5%) vs. ~0.5 in ice, consistent with the general trend found with our 18S sequencing (Supplementary Fig. 2a, b). We also quantified prokaryotic PGs (with bacterial taxa identified via 16S amplicon sequencing, Supplementary Fig. 2c, d), as well as metazoan and other classified and unclassified PGs in both sample types (Fig. 1c, d). One amoebozoan PG was identified in ice, and two viral PGs were found in snow. We subsequently filtered for all chlorophyte PGs in snow and streptophyte PGs in ice to compare their functional proteome composition, and hereafter we refer to these as the chlorophyte proteome and the streptophyte proteome, with individual PGs expressed relative to the total amount of weighted spectra for each proteome.

### Primary metabolism, regulation of gene expression, and environmental signaling

**Fig. 2:**
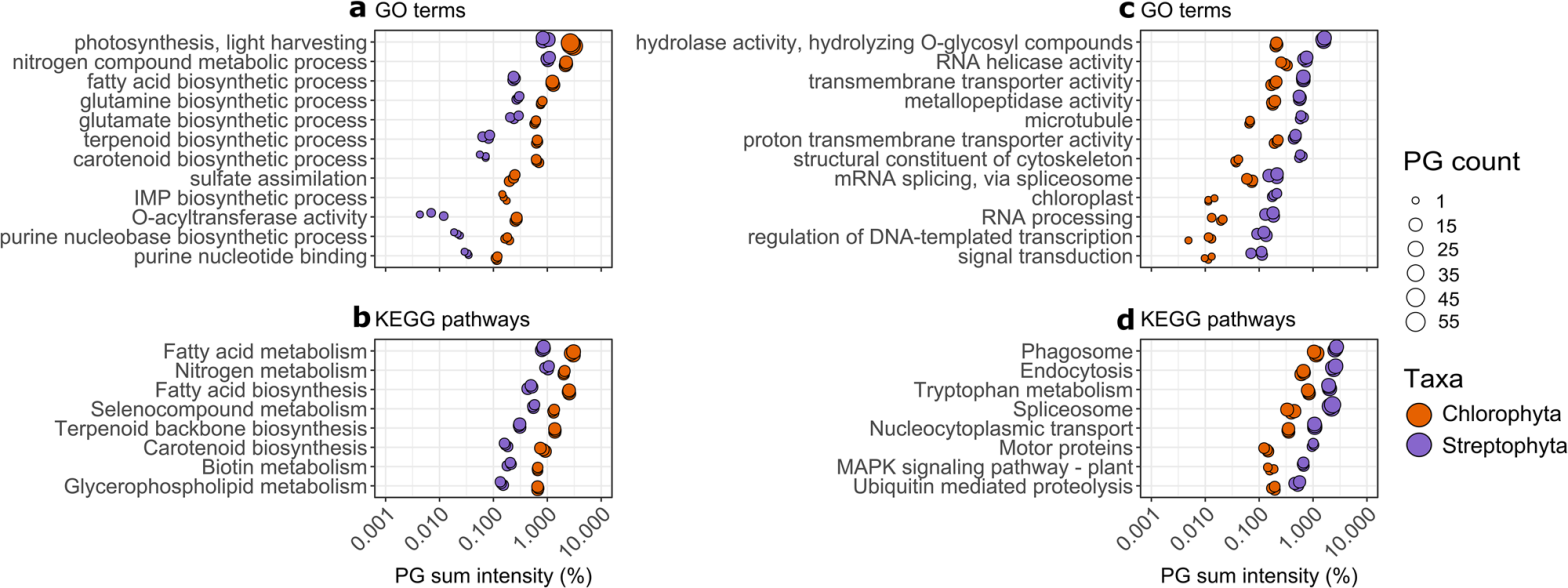
Selected protein group GO terms and KEGG pathways. Data includes pathways **a, b** enriched in the chlorophyte proteome (*t*-tests, *p*val < 0.05, log10 FC > 0.3) and **c, d** enriched in the streptophyte proteome (*t*-tests, *p* < 0.05, log10 FC > 0.3). The number of PGs associated with each functional group is represented by the size of the symbol. The full set of KEGG and GO terms is provided in Supplementary Data 2 and 3. All three replicates for each sample are shown with the same symbol to ease comparison of symbol size. PG protein group, FC Fold change, IMP inosine monophosphate, MAPK mitogen-activated protein kinases. All acronyms and abbreviations used in this figure are explained in Supplementary Data 4.

We annotated all protein sequences with GO terms^31,32^, KEGG pathways^33^, and INTERPRO^34^ domains, allowing a functional comparison between proteomes. Between the chlorophyte and streptophyte proteomes, the relative abundance of high-abundance, essential functional groups such as the GO terms *ATP binding* and *translation*, and the KEGG pathways *Biosynthesis of secondary metabolites* and *Carbon metabolism* (Supplementary Figs. 4a, b and 5, Supplementary Data 2 and 3) did not significantly differ. Only a few of the quantified functional groups differed between the two taxonomic groups (Fig. 2, Supplementary Data 2 and 3, Supplementary Fig. 4a, b).

**Fig. 3:**
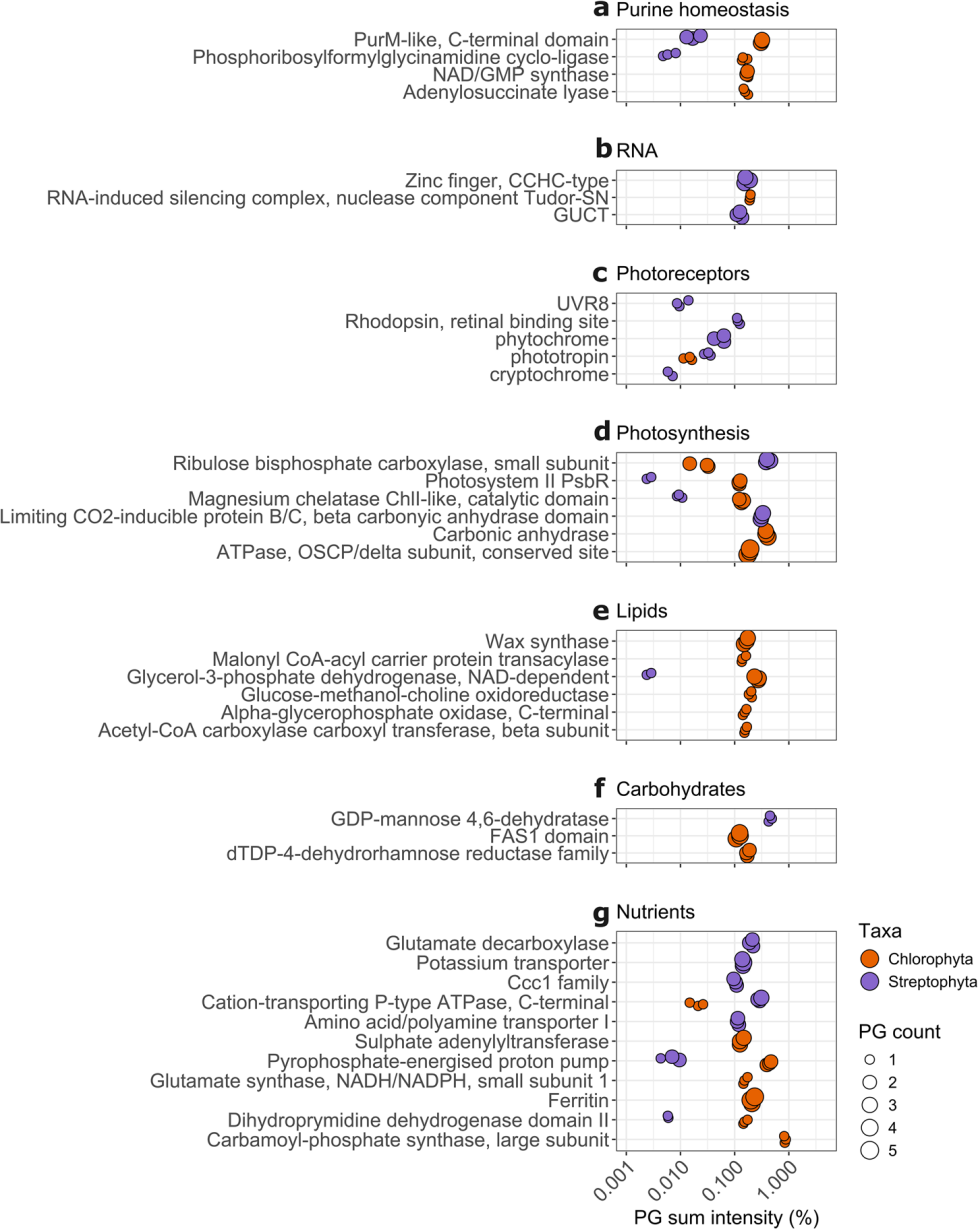
Protein group functions with significantly different abundance in the chlorophyte vs. the streptophyte proteome. Plotted PGs are associated with **a** purine homeostasis, **b** RNA, **c** photoreceptors, **d** photosynthesis, **e** lipids, **f** carbohydrates, and **g** nutrients. Functional groups are expressed as the percentage of PGs with such an annotation in each proteome. Functions were assigned with INTERPRO domains, except for phytochrome, phototropin, UVR8, and cryptochrome, which were identified by reciprocal BLASTp. The number of proteins associated with each function (PG count) is represented by the size of the symbol. All protein functions shown are significantly enriched in one proteome (*p* < 0.05, Log10 FC > 1, PG sum intensity > 0.1% in the sample enriched in the annotation, see “Methods” and Supplementary Fig. 4c, except for protein identified by reciprocal BLASTp). All three replicates for each sample are shown with the same symbol to ease comparison of symbol size. PG protein group, FC Fold change, PurM aminoimidazole ribonucleotide synthetase, CCHC-type CysCysHisCys-type, Tudor-SN Tudor staphylococcal nuclease, GUCT Gu C-terminal domain, UVR8 UV-B resistance 8, PsbR Photosystem II subunit R, OSCP oligomycin sensitivity conferral protein, FAS1 fasciclin-like, Ccc1 cross-complements Ca2+ phenotype of csg1, P-type phosphorylation-type. All acronyms and abbreviations used in this figure are explained in Supplementary Data 4.

Purine synthesis PGs were more abundant in the chlorophyte proteome with the GO terms *purine nucleobase biosynthetic process* (~10× more abundant) and the *purine nucleotide binding* and *IMP biosynthetic process* (Fig. 2a), as well as functional domains (assigned with INTERPRO) such as *NAD/GMP synthase* and *Adenylosuccinate lyase* also being at least 10× more abundant (Fig. 3a). The metabolomes of the same samples (see “Methods”) revealed that indeed the purines hypoxanthine and adenine accounted for ~4% and 2% of the snow metabolome, respectively, but much less than 1% each in the ice metabolome (Supplementary Fig. 6a). In contrast, PGs linked to RNA metabolism were enriched in the streptophyte proteome, indicated by an increase in PGs annotated with GO terms *RNA helicase activity* and *RNA processing*, and the KEGG pathway *Spliceosome* (Fig. 2c, d). Furthermore, individual PG annotations revealed an enrichment in *GUCT* domain-containing proteins typical for certain RNA helicases (Fig. 3b). We also found a high abundance of one PG (>0.1%) with the annotation *RNA-induced silencing complex, nuclease component Tudor-SN* in the chlorophyte proteome, homologous to a plant protein known to inhibit the degradation of specific mRNAs in response to stress^35^ (Fig. 3b). The streptophyte proteome also had a notable abundance of PGs linked to protein degradation (with GO term *metallopeptidase activity* and KEGG pathway *Ubiquitin mediated proteolysis)* and individual PG annotations (including the FtsH protein, involved in the turnover of photosystem II proteins; Fig. 2c, d, Supplementary Fig. 7a). PGs involved in endocytosis, and phagocytosis^36^ were also enriched in the streptophyte proteome (Fig. 2c, d). The PGs with the GO terms *structural constituent of cytoskeleton* and *microtubule* were almost ~10× more abundant in the streptophyte proteome (Fig. 2c), largely due to the contribution of tubulin (Supplementary Fig. 7b).

Our functional analysis also indicated that PGs linked to signal transduction were more abundant in the streptophyte proteome, with the enrichment of the GO terms *regulation of DNA-templated transcription* (over ~10× more abundant) and *signal transduction*, and the KEGG pathway *MAPK signaling pathway–plant* (~6× more abundant; Fig. 2c, d). We identified numerous transcription factors (TFs) in both proteomes (Supplementary Fig. 8a), including Cold Shock Domain TFs, with the TF annotation *Zinc finger, CCHC-type* enriched in the streptophyte proteome. One PG with an ice-binding domain was quantified in an ice sample, homologous to the *Ancylonema* ice-binding protein identified by Procházková et al.^37^. However, this PG could not be classified beyond “cellular organisms” by MEGAN (accession 149312, Supplementary Data 1). Using reciprocal BLASTp, we identified multiple photoreceptors, including the UV-sensing UVR8 PG in the streptophyte proteome (Fig. 3c). A high-abundance light-sensitive rhodopsin PG (identified with INTERPRO) also represented >0.1% of the streptophyte proteome (Fig. 3c). The chlorophyte proteome had only one photoreceptor: phototropin.

### Photosynthesis, carbon partitioning, and nutrient homeostasis

The most abundant protein in all samples was the large RuBisCO subunit (rbcL), invariably classified as chlorophyte in the snow samples and streptophyte in the ice sample (Supplementary Fig. 9). Overall, photosynthetic PGs were not found to be enriched in either proteome (Supplementary Fig. 5a, b). Both proteomes had high abundances of different carbonic anhydrases (annotations *Carbonic anhydrase* and *Limiting CO2-inducible protein B/C, beta carbonyic anhydrase domain*) involved in the conversion of carbon dioxide into bicarbonate (Fig. 3d). Based on GO term analysis, *Photosynthesis, light harvesting* PGs, made up of Chlorophyll A-B binding proteins were over three times more abundant in the chlorophyte proteome (Fig. 2a), with other photosynthesis PGs also found to be ~10× more abundant in the chlorophyte proteome (Fig. 3d). In contrast, the streptophyte proteome had ~10× more RuBisCO small subunit (rbcS) PGs and PGs associated with the *chloroplast* GO term (Figs. 2c and 3d). Finally, total Reactive Oxygen Species homeostasis PGs with the GO term *oxidoreductase activity* did not significantly differ between proteomes (Supplementary Fig. 5a), although individual PG functions for oxidative stress response differed between taxa (Supplementary Fig. 7c).

Proteins involved in lipid and fatty acid metabolism were more abundant in the chlorophyte proteome, as indicated by the GO terms *fatty acid biosynthetic process* and *O-acyltransferase activity*, as well as the KEGG pathways *Fatty acid metabolism*, *Biotin metabolism*, and *Glycerophospholipid metabolism* (Fig. 2a, b). PGs with a *Glycerol-3-phosphate dehydrogenase NAD-dependent* domain were ~100× more abundant in the chlorophyte proteome (Fig. 3e). The chlorophyte proteome also had a >0.1% abundance of the putative mitochondrial glycerol-3-phosphate dehydrogenase activity (*Alpha-glycerophosphate oxidase, C-terminal*) (Fig. 3e). Glycerol-3-phosphate was also noticeably more abundant by a similar order of magnitude in the snow metabolome, with no clear difference in the amount of cellular glycerol (Supplementary Fig. 6b). The chlorophyte proteome also contained highly expressed PGs annotated as *Wax synthase* and *Glucose-methanol-choline oxidoreductase*, all linked to lipid and fatty acid metabolism (Fig. 3e). Glucose-methanol-choline oxidoreductases have been linked to hydrocarbon production in algae and photosynthetic efficiency in the cold^38^. However, the snow metabolome was not enriched in the hydrocarbon metabolites covered in our analysis (Supplementary Fig. 6c). In terms of carbohydrate synthesis, a PG with the annotation *GDP-mannose 4,6 dehydratase*, involved in the production of fucose^39^, was highly abundant (~0.5%) in the streptophyte proteome. In contrast, two PGs with the annotation *dTDP-4-dehydrorhamnose reductase family*, linked to the production of rhamnose^40^, constituted 0.2% of the chlorophyte proteome (Fig. 3f). While a difference in fucose abundance in the metabolite data was not evident, we found that rhamnose was ~10× more enriched in the snow metabolome (Supplementary Fig. 6d). Four PGs with a *FAS1 domain* annotation represented 0.1% of the chlorophyte proteome, which are predicted to have a role in extracellular cell adhesion^41^ or be homologous to the chlorophyte water soluble astaxanthin-binding carotenoprotein^42^ (Fig. 3f). In terms of polysaccharide degradation, we found that over 1.5% of the streptophyte proteome was made up of glycosyl hydrolases responsible for the hydrolysis of O-glycosyl compounds (compared to 0.2% for the chlorophyte proteome; Fig. 2c).

PGs linked to organic and inorganic nutrient homeostasis were found in both proteomes. Both taxa were characterized by a similar abundance of transmembrane proteins (~10% of both proteomes, Supplementary Fig. 8b). However, we found a high PG abundance of the GO term *transmembrane transporter activity* in the streptophyte proteome (~0.65% of proteome, vs. ~0.2% in the chlorophyte proteome; Fig. 2c). Such enriched proteins included those with domains involved in the transport of inorganic ions (i.e., *Potassium transporter* and *Ccc1 family*) or organic compounds (i.e., *Amino acid/polyamine transporter I*; Fig. 3g). Proteins involved in nitrogen metabolism were enriched in the chlorophyte proteome, mainly linked to glutamine and glutamate (glutamic acid) synthesis (Figs. 2a and 3g). Our metabolite data showed that glutamate, glutamine, and pyroglutamic acid all contributed ~10× more to the chlorophyte metabolome (Supplementary Fig. 6e). Snow algae had an enrichment of the annotation *Dihydropyrimidine dehydrogenase domain II*, linked to pyrimidine breakdown. Consistent with these metabolome results, PGs with the domain *Glutamate decarboxylase*, responsible for the conversion of glutamate to γ-aminobutyrate, were only present in the streptophyte proteome (Fig. 3g). Further striking differences between the two proteomes include, for example a higher PG abundance linked to *Tryptophan metabolism* (Fig. 2d) in the streptophyte proteome, while the chlorophyte proteome had a large amount of ferritin PGs (ferritin PGs were quantified in the ice samples but were not associated to streptophyte algae; Fig. 3g, Supplementary Data 1). The chlorophyte proteome had a high abundance of PGs with a *Pyrophosphate-energized proton pump domain* (over 60x more abundant; Fig. 3g), as well as PGs linked to sulfur metabolism with the GO term *sulfate assimilation* (Fig. 2a), including three PGs with the domain *Sulphate adenylyltransferase* (Fig. 3g).

### Pigment production pathways

**Fig. 4:**
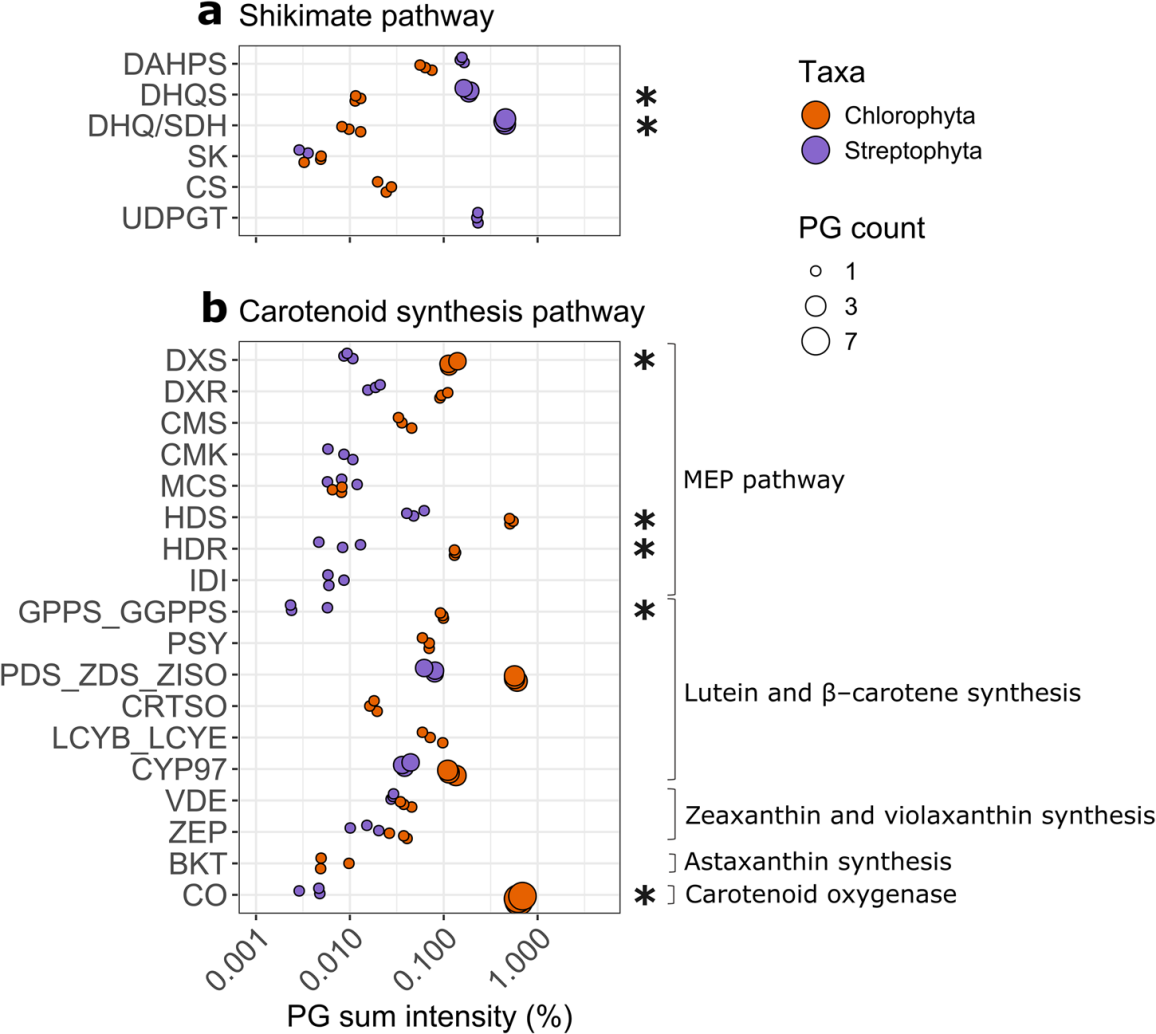
Pigment biosynthesis pathways in both proteomes. PGs plotted are involved in **a** the shikimate pathway and possible purpurogallin production, **b** the carotenoid synthesis pathway. Functional groups are expressed as the percentage of proteins with such a function in each proteome. Functions were assigned with reciprocal blastp and INTERPRO annotations. The number of proteins associated with each function (PG count) is represented by the size of the symbol, symbolized by protein functions quantified in both proteomes, significantly enriched functions in one proteome (*t*-test, *p* < 0.05, Log10 FC > 1) are annotated with *. All three replicates for each sample are shown with the same symbol to ease comparison of symbol size. PG protein group, MEP methylerythritol phosphate pathway, FC fold change, DAHPS 3-Deoxy-D-arabinoheptulosonate 7-phosphate synthase, DHQS 3-dehydroquinate synthase, DHQ/SDH bifunctional 3-dehydroquinate dehydratase/shikimate dehydrogenase, SK shikimate Kinase, CS chorismate synthase, UDPGT UDP-glucosyltransferases, DXS 1-deoxy-d-xylulose 5-phosphate synthase, DXR 1-deoxy-D-xylulose 5-phosphate reductoisomerase, CMS 2-C-methyl-d-erythritol, CMK 4-diphosphocytidyl-2-C-methyl-d-erythritol kinase, MCS 2-C-methyl-d-erythritol 2,4-cyclodiphosphate synthase, HDS 4-hydroxy-3-methylbut-2-en-1-yl diphosphate synthase, HDR 4-hydroxy-3-methylbut-2-en-1-yl diphosphate reductase, GPPS_GGPPS geranyl diphosphate synthase/geranylgeranyl diphosphate synthase, IDI isopentenyl-diphosphate Delta-isomerase I, PSY phytoene synthase 1, PDS_ZDS_ZISO 15-cis-phytoene desaturase/Zeta-carotene desaturase/15-cis-zeta-carotene isomerase, CRTSO 4-phosphate cytidylyltransferase, LCYB_LCYE lycopene beta cyclase/Lycopene epsilon cyclase, CYP97 cytochrome P450 epsilon hydroxylase_cytochrome P450 beta hydroxylase, VDE violaxanthin de-epoxidase, ZEP zeaxanthin epoxidase, BKT beta-carotenoid ketolase, CO carotenoid oxygenase. All acronyms and abbreviations used in this figure are explained in Supplementary Data 4.

The primary photoprotective pigment produced by *Ancylonema* spp., purpurogallin, is a hydrolysable tannin^6^. The synthesis pathway of purpurogallin has yet to be described, but the early steps of the shikimate pathway may be essential for its production^22^. We identified enzymes of the shikimate pathway using reciprocal BLASTp, and found that most proteins were present in both proteomes (Fig. 4a). We found a >10× higher relative abundance of the second (DHQS, 3-dehydroquinate synthase) and third (DHQ/SDH, bifunctional 3-dehydroquinate dehydratase/shikimate dehydrogenase) enzyme of this pathway in the streptophyte proteome (Fig. 4a). We also document the high expression of one UDP-glucosyltransferases (UDPGT) in the streptophyte proteome at a similar abundance to DHQS and DHHQD/SD (Fig. 4a). UDPGTs are essential in the production of hydrolysable tannins in other plant species^43^. Conversely, for the carotenoid pigments typical of chlorophyte snow algae, both GO and KEGG analyses indicated an enrichment for PGs involved in terpenoid and carotenoid biosynthesis in the chlorophyte proteome (Fig. 2a, b). Reciprocal BLASTp identified multiple PGs belonging to the terpenoid synthesis methylerythritol phosphate pathway and carotenoid synthesis in both proteomes, with many >10× more abundant in the chlorophyte proteome (Fig. 4b). PGs involved in carotenoid cleavage and the production of apocarotenoids, Carotenoid Oxygenases, were ~100× more abundant in the chlorophyte proteome (Fig. 4b). In contrast, the only apocarotenoid quantified in the metabolome, a Vitamin A fatty acid ester^44^, was not enriched in the snow metabolome (Supplementary Fig. 6f).

## Discussion

We quantified proteins from biota on the GrIS, allowing us, for the first time, to disentangle key, previously inaccessible, biological traits of chlorophyte snow and streptophyte glacier ice algae linked to important environmental variables of the GrIS, such as high light and low nutrient availability. The relative protein abundances of the different dominant taxa (Fig. 1) match the phyla of previously described red snow and dark ice microbiomes, based on nucleic acid sequencing and microscopy, both on the GrIS and globally^1,3–5,9,10,14,15,45^, validating our metaproteomics workflow. Using protein as a proxy for biomass^46^, this workflow provides a first possibility to distinguish the relative contributions of different taxa to the total biomass in snow and ice samples.

Life on the GrIS during the summer melt months involves survival under extremely high light; however, both algal taxa continue to be photosynthetically active (Supplementary Note 1). The higher expression of photoreceptors likely indicates that the streptophyte algal cells are able to tune their cellular physiology to variations in light intensity and availability (Fig. 3c). This could include optimizing the position of their chloroplasts under the prevailing solar radiation, either for photosynthesis or for photoprotection^47^, as has been studied in other zygnematophytes^48^. Furthermore, the higher abundance of proteins linked to signal transduction in the streptophyte proteome (Fig. 2c, d) suggests that *Ancylonema* spp. are better at sensing and responding to environmental signals than the snow algae. The only photoreceptor quantified in the snow algae proteome, phototropin (Fig. 3c), has been linked to zygote formation in the related mesophilic chlorophyte *Chlamydomonas reinhardtii*, with an essential role in blue light sensing for the regulation of algal life cycle^49^. The role of blue light sensing could be a conserved feature of hypnozygote formation in chlorophyte snow algae.

The GrIS is a typical oligotrophic environment^16^, and nutrients steadily become depleted after the initial snowmelt at the beginning of the bloom season. The quantification of multiple nutrient transport proteins provides an indication that the streptophyte algae are prepared to transport and use dissolved nutrients (e.g., K, Mn, amino acids) once they become available (Figs. 2c and 3g). The high abundance of PGs linked to RNA and protein breakdown in the streptophyte algae could be linked to optimized recycling of cellular material (both molecules are rich in organic phosphorus^50^), in particular for damaged or unused molecules. Such adaptations would allow *Ancylonema* spp. to sustain cell division rather than form a cyst stage like the snow algae. Snow algae are widely known to be nitrogen-limited^51^, with nitrogen limitation being a trigger for secondary carotenoid production^52^. Our proteomic data is consistent with an important role for nitrogen metabolism in our chlorophyte snow algae, in particular those linked to purine and glutamine/glutamate as possible nitrogen storage molecules (Figs. 2a and 3a, g). The recent discovery of crystalline guanine as a nitrogen store in algal cells^53^ substantiates this claim, and could be linked to unidentified crystal content in snow algae vacuoles^54,55^. This stored nitrogen could originate from early melt season nutrient leaching from snow as nutrients fractionate from snow crystals into snowmelt.

Both algal taxa produce pigments that darken the surfaces of snow and ice^13^. Equally, both are likely to use a large proportion of their available organic carbon pools for the synthesis of such photoprotective pigments, as these pigments exist in much higher cellular concentrations than chlorophyll^17,18^. The high cellular pigment abundance is reflected in the enzyme abundance of the pathways responsible for synthesizing them (Fig. 4). For *Ancylonema*, the high abundance of early shikimate pathway proteins, including DHQ/SDH, concurs with the hypothesis derived from recently published genomic data that early shikimate pathway enzymes have higher gene copies to increase pathway activity with higher enzyme abundance^22^. The high expression of PGs with O-glycosyl hydrolase activity (Fig. 2b) in the streptophyte proteome may also indicate that these algae are actively breaking down their own purpurogallin. Light stress could lead to spontaneous pigment degradation, and cells may need to maintain continuous synthesis/breakdown of purpurogallin to optimize the high abundance of intact molecules.

Red snow algae cells contain large amounts of astaxanthin esters^4,14,17^, quantified in our protein data, through an enrichment of chlorophyte PGs involved in terpenoids and carotenoids production (Fig. 4b). Similarly, the high abundance of PGs involved in lipid synthesis in the chlorophyte proteome (Fig. 3e), consistent with a previous report that snow algae have more lipids than glacier ice algae^56^, can also be linked to carotenoid esterification. Our proteome data show that the enzymes involved in the upstream steps of astaxanthin production are more abundant than those directly involved in astaxanthin production from *β*-carotene. While other unidentified enzymes may control astaxanthin production from *β*-carotene, it is also possible that unused *β*-carotene is broken down into apocarotenoids by carotenoid oxygenases. Apocarotenoids can be volatile, and while no apocarotenoid was quantified in the GrIS volatilome^57^, the expression of these proteins could be the molecular basis of the previously reported sweet smell associated with late bloom red snowfields^58^.

These first GrIS metaproteomes identify important, and previously inaccessible, clues regarding the adaptation of snow and glacier ice algae to the extreme conditions of the GrIS. Our protein data indicate that streptophyte glacier ice algae are more in tune with characteristics of their ice-matrix environment, including photodamaging high light and oligotrophy, with more proteins linked to light perception and the uptake of much-needed, yet scarce nutrients. In contrast, chlorophyte snow algae protein profiles are characterized by lipid and carotenoid synthesis, with strong indications of nitrogen limitation. This information can serve as fundamental building blocks for further experimental work on these algal taxa, for example, investigating a role in pigment breakdown for glycosyl hydrolases in *Ancylonema* spp. and carotenoid oxygenases in snow algae. Pigment breakdown could be ecologically very relevant by providing an essential energy source during the burial of organisms under snow during winter.

## Methods

### Sample description and collection

Snow and ice samples with high algal biomass were collected from the GrIS in July and August 2021 in south Greenland near Narsarsuaq^10,13,19^ (61°05′ N,46°50′ W, Supplementary Fig. 1a), as part of the DEEP PURPLE ERC (https://www.deeppurple-ercsyg.eu/about) ice camp DP21. Red snow samples were collected on the 24th July, and dark ice on the 7th August. Dark ice and red snow were collected using a pre-conditioned ice axe (for ice) and trowel and stored in sterile whirl-pak^®^ bags to melt. Samples for DNA, protein, and cell counts were melted at ambient temperature (5 °C), with one bag for red snow and three bags for dark ice. Approximately 100 mL of samples for DNA (1 filter for red snow, 3 filters for dark ice) and protein (three filters for snow and three filters for ice) were filtered on sterile 0.2 µm cellulose nitrate filters (Sartorius), and frozen at −80 °C in a portable freezer (Sterling ULT25), transported from Greenland to Potsdam (Germany) using a cryoshipper and subsequently stored until extraction at −80 °C. For intracellular metabolites, samples were collected in separate bags, five for snow, three for ice. For each bag, 45 mL aliquots of sample were filtered in triplicates or quadruplicates (totaling 19 aliquots for red snow and 9 aliquots for dark ice) on pre-combusted glass fiber filters (25 mm, 0.7 µm pore size, Whatman), quenched with 50% methanol in 2 mL Eppendorf tubes, and stored and transported at −20 °C until extraction^19^. Photographs of typical red snow and dark ice sampling sites (Supplementary Fig. 1b, d) are shown together with light microscopy images (100 fold magnification, VisiScope100, Model BL124) of the dominant algae in each sample type (Supplementary Fig. 1c, e). Approximately 12 mL of each sample was fixed with 2.5% glutaraldehyde and stored at 4 °C for cell counts.

### Generation of predicted protein database

Protein sequences translated from previously sequenced polyA-isolated metatranscriptomes (Illumina-HiSeq paired-end short reads, 2 × 150 bp) from cryoconite, snow, and ice samples of the GrIS were used as a database for protein identification. The samples were collected during previous field seasons (in 2019 and 2020) and are not the same as the protein samples presented in this study. The sequencing information has been described previously by Perini et al.^11^. Our current study used the following RNA sequencing samples: MG3, MG5, MG6, MG7, MG8, MG11, MG12, MG14, MG19, MG22, MG23, MG24, MG25, MG26. MG27, MG28, MG30, MG31 from that paper. To generate a predicted protein database, these sequences were assembled with TRINITY^59^, and open reading frames were identified with PRODIGAL^60^, generating a database of 437,423 predicted protein sequences after dereplication at 100% with CD-HIT^61^.

### Protein extraction and digestion

Proteins were extracted from filters following a similar method to Deusch and Seifert^62^. Briefly, frozen filters were broken up using clean scissors and tweezers and transferred to a 15 mL Falcon tube. Filter pieces were submerged in 1 mL of a first buffer (50 mM Tris-HCl pH 7.5, 0.1 mg/mL chloramphenicol, 1 mM PMSF), and vortexed at the highest speed for 10 s. 1.5 mL of a second buffer (20 mM Tris-HCl pH 7.5 and 2% SDS) was added, and samples were vortexed again for 10 s at the highest speed. Samples were incubated at 60 °C at 1000 rpm (Eppendorf, ThermoMixer C), before the addition of 5 mL of a third buffer (20 mM Tris-HCl pH 7.5, 0.1 mg/mL MgCl_2_, 1 mM PMSF, and 1 µg/mL DNase I, PanReac AppliChem). Samples were sonicated 2 × 3 min in an ice-cold ultrasonication bath, homogenized by inverting the tubes, and subsequently again incubated at 37 °C at 1000 rpm for 10 min. Filter pieces and other particulates (including minerals) were pelleted by centrifugation at 4000 × *g* (1 min, 4 °C, Eppendorf), and the protein-containing supernatant was transferred to a new 15 mL Falcon tube. This centrifugation step was repeated once more, before protein precipitation with trichloroacetic acid (final concentration 20% v/v, MP Biomedicals) for 20 min on ice, followed by centrifugation at 12,000 × *g* for 30 min (4 °C, SIGMA). The pellet containing the protein was subsequently washed 3 times with LC-MS grade ice-cold acetone (Roth) by centrifugation at 12,000 × *g* for 10 min 4 °C. Pellets were air dried to remove any remaining acetone, and protein was solubilized in 2x Laemmli buffer (Omnilab) by pipetting and sonication and subsequently stored frozen at −80 °C until further use.

For 1D-SDS-PAGE, 3.7% 2-mercaptoethanol (v/v) (freshly prepared before use) was added to the protein samples, incubated for 5 min at 98 °C before additionally incubating it in an ultrasonication bath for 5 min and separating via SDS-PAGE (Criterion TG 4–20% Precast Midi Gel, BIO-RAD Laboratories, Inc., USA). After fixation and staining with Coomassie, each gel lane was cut into 10 pieces as previously described with some modifications^63^. Briefly, gel pieces were destained 3 times for 15 min with 1 mL of gel washing buffer (200 mM ammonium bicarbonate in 30% acetonitrile (v/v)) at 37 °C under vigorous shaking and subsequently dehydrated in 1 mL 100% acetonitrile (v/v) for 15 min. After dehydration, the supernatant was removed and the gel pieces were dried in a vacuum centrifuge (Eppendorf) at 30 °C for 20 min. Proteins were in-gel reduced by adding 100 µL 10 mM dithiothreitol in 25 mM ammonium bicarbonate buffer (1 h at 56 °C) and alkylated (after cooling at room temperature) with 100 µL 55 mM iodoacetamide in 25 mM ammonium bicarbonate buffer (in the dark for 45 min at room temperature) before the supernatant was removed. The gel pieces were washed with 1 mL 25 mM ammonium bicarbonate buffer (10 min, 900 rpm at room temperature), and dehydrated with 500 µL 100% acetonitrile for 10 min. The supernatant was removed before gel pieces were dried in a vacuum centrifuge (30 min), and covered with 120 µL trypsin solution (2 µg/mL Trypsin, Promega™). After rehydration, the remaining supernatant was removed before the samples were incubated in a thermo-mixer (Eppendorf) for 14 h at 37 °C without shaking. Peptides were eluted with 120 µL water (ASTM Type 1) by sonication for 15 min before the peptide-containing supernatant was transferred into a new tube. The peptides were dried down completely in a vacuum centrifuge and then reconstituted in 15 µL solvent A (0.1% acetic acid; v/v). The peptides were desalted via ZipTips C18 (Merck Millipore, P10 tip size) according to the manufacturer’s protocol. The eluted samples were dried in a vacuum centrifuge and resuspended in 10 µL solvent A.

### Liquid chromatography with tandem mass spectrometry (LC-MS/MS) analysis

For LC-MS/MS measurement, peptides were analyzed on an LTQ Orbitrap Elite instrument (ThermoFisher Scientific, Waltham, MA, USA) coupled to an EASY-nLC 1200 liquid chromatography system. Therefore, peptides were loaded onto in-house packed capillary columns (20 cm length, 100 µm inner diameter) filled with Dr. Maisch ReproSil Pur 120 C18-AQ 3 µm (Dr. Maisch GmbH, Ammerbuch-Entringen, Germany) in a one-column setup at a constant temperature of 45 °C and separated using an 83 min nonlinear binary gradient from 5% to 99% solvent B (95% acetonitrile (v/v), 0.1% acetic acid (v/v)) in solvent A (0.1% acetic acid (v/v)) at a constant flow rate of 300 nL/min. In the Orbitrap, the MS1 scan was recorded with a mass window of 300–1700 *m*/*z* and a resolution of 60,000. The 20 most intense precursor ions were selected for collision-induced dissociation. Ions with an unassigned charge or a charge of 1, or >8 are excluded. Dynamic exclusion and lock mass correction were enabled.

### Protein identification

All MS/MS spectra were analyzed using Mascot^64^ (version 2.7.0.1; Matrix Science, London, UK) and searched against the predicted protein database containing 824,320 entries (including forward and reverse entries as well as common lab contaminations), assuming the digestion enzyme trypsin. For the database search with Mascot, the following parameters were used: fragment ion mass tolerance of 0.5 Da and parent ion tolerance of 10 ppm, no missed cleavages, variable modification on methionine (oxidation), and fixed modification on cysteine (carbamidomethylation). Scaffold^65^ (version 5.2.2; Proteome Software Inc., Portland, OR) was used to merge the search results and to validate MS/MS-based peptide and protein identifications. During the creation of the Scaffold file, an additional X! Tandem search was performed for validation (version 2017.2.1.4; The GPM, thegpm.org; version X! Tandem Alanine)^66^ with default settings (fragment ion mass tolerance of 0.5 Da and parent ion tolerance of 10 ppm, carbamidomethyl on cysteine as fixed modification, Glu->pyro-Glu of the n-terminus, ammonia-loss of the n-terminus, Gln->pyro-Glu of the n-terminus and oxidation on methionine as variable modifications). Peptide identifications were accepted if they could be established at greater than 95% probability by the Peptide Prophet algorithm^67^ with Scaffold delta-mass correction. Protein identifications were accepted if they could be established at greater than 99% probability and contained at least 2 identified peptides. Protein probabilities were assigned by the Protein Prophet algorithm^68^. Proteins that contained similar peptides and could not be differentiated based on MS/MS analysis alone were grouped as PGs to satisfy the principles of parsimony. For (semi-)quantitative analysis, the Scaffold’s “Quantitative Value” (by multiplying each spectra value by the average spectra over all samples/total spectra in each sample) for normalized weighted spectra for each PG was used. Proteins were identified with at least two unique peptides in at least one biological sample, and proteins were quantified with at least two unique peptides in at least two of the three biological replicates. The mass spectrometry proteomics data have been deposited to the ProteomeXchange Consortium (http://proteomecentral.proteomexchange.org) via the PRIDE partner repository with the dataset identifier PXD057047.

### Protein sequence annotation

Taxonomic annotation of master proteins, a representative protein for each PG sequence, was undertaken by DIAMOND v0.8.22 BLASTp^69^ and MEGAN6 v6.24.23^70^. DIAMOND BLASTp was run against the NCBI nr databases (downloaded 05.06.2023), with parameters: –evalue 1e-5 and --top 500. Protein taxonomy was assigned using a Last Common Ancestor (LCA) approach in MEGAN6 with the following parameters: Min Score: 50, Max Expected: 0.0001, Min Percent Identity: 0, Top Percent Identity: 10.0, Min Support Percent: 0.01, Min Support: 0, Min read Length: 0, LCA Algorithm: weighted, Percent to Cover: 80, using a MEGAN map from February 2022. The number of protein sequences in the predicted protein database linked to different taxonomic groups is available in Supplementary Table 1.

For downstream quantitative analysis, we calculated the sum % for each PG within a given sample by dividing the normalized weighted spectral value of each PG by the sum of all normalized weighted spectral values within a sample. Furthermore, we extracted semi-quantitative information about algal PGs specifically. To do this, we extracted all chlorophyte proteins from the snow samples and all streptophyte proteins from the ice samples, and divided each protein by the sum of all proteins from each respective group, generating a chlorophyte and a streptophyte proteome in protein percentages.

Functional annotations of proteins were undertaken using INTERPROSCAN v5.65^34^, assigned GO terms (Supplementary Data 1–3) with interpro2GO, and KEGG numbers were assigned with GhostKOALA^33^, and were then subsequently assigned KEGG Pathway. For the chlorophyte and streptophyte proteomes, to filter out any non-Viridiplantae pathways, we only kept pathways that are described for *Chlamydomonas reinhardtii* (https://www.genome.jp/kegg-bin/show_organism?menu_type=pathway_maps&org=cre) and/or *Arabidopsis thaliana* (https://www.genome.jp/kegg-bin/show_organism?menu_type=pathway_maps&org=ath). We considered GO and KEGG pathways to be enriched in a proteome if the log10 fold change was ≥0.3, the sum percentage PG intensity in the enriched proteome was ≥0.1%, and the *t*-test *p* ≤ 0.05. We considered INTERPRO annotations to be enriched in a proteome if the log10 fold change was ≥1, the sum percentage PG intensity in the enriched proteome was ≥0.1%, and the *t*-test *p* ≤ 0.05. Data showing all comparative enrichments and cut-off values for GO terms, KEGG pathways, and INTERPRO annotations are plotted in Supplementary Fig. 4.

TF proteins were identified with iTAK^71^, and transmembrane proteins with TMHMM-2.0^72^. We used reciprocal BLASTp to identify shikimate pathway proteins, photoreceptors, and MEP/Carotenoid proteins using DIAMOND with a maximum evalue of 1e-5, minimum sequence coverage of 60%, and minimum ID 30%. Shikimate pathway proteins were identified using reviewed proteins from Uniprot^73^ for *Arabidopsis thaliana*, and previously identified proteins for *Chlamydomonas reinhardtii* and *Klebsormidium nitens*. Photoreceptor proteins were from reviewed proteins on uniport for *A. thaliana* and from published protein accessions for *C. reinhardtii*^74^. MEP/Carotenoid pathway proteins were searched against published *C. zofingiensis*^75^ and reviewed *A. thaliana* proteins^76^.

### DNA extraction and sequencing

DNA was extracted and analyzed following the same method outlined in Peter et al.^19^. DNA was amplified with prokaryotic primers for the 16S rRNA gene Bakt_341F (5′-CCTAYGGGRBGCASCAG-3′) and Bakt_805R (5’-GGACTACNNGGGTATCTAAT-3’), and with the eukaryotic primers for the 18S rRNA gene using 528F (5′- GCGGTAATTCCAGCTCCAA-3′) and 706R (5′-AATCCRAGAATTTCACCTCT-3′). The targeted marker genes’ raw sequencing data were pre-processed using the DADA2 (vers 1.16) R (4.2.2) package^77^ for the removal of primers, non-target length, and chimera sequences, and for merging the reads. Sequences tables were merged, and taxonomy was assigned for 16S and 18S rRNA gene sequences using the SILVA v138 database^78^. Taxa which were not assigned at the genus level were assigned to the upper taxonomic rank to be considered using the function tax_fix() from the package microViz^79^ (0.10.8). Certain taxa not assigned to any genus with this method were subjected to a manual BLAST. Data was assembled and processed in the form of a Phyloseq^80^ (vers. 1.42.0) object. Only one replicate of red snow was sequenced, while the ice samples were sequenced in triplicates, and the data were averaged across replicates for plotting. Full sequencing data is shown in Supplementary Fig. 2a, d.

### FlowCam algae cell counts and biovolume measurements

Cell counts were acquired using a FlowCam 5000 (Yokowaga Fluid Imaging Technologies, Inc., Scarborough, ME, USA), equipped with a 10× objective, and the associated Visual Spreadsheet software (v5.9.0.74, Yokogawa Fluid Imaging Technologies, Inc., Scarborough, ME, USA), following a similar protocol to Peter et al.^19^. Each measurement was done with a flow rate of 0.250 mL/min, the distance to the nearest neighbor set to 0.1 µm, a pixel intensity threshold for both dark and white pixels set to 15, and 5 close hole iterations. The auto image rate was chosen to match the highest possible theoretical efficiency of 71%. Each sample, one snow sample and three ice samples, was measured in technical triplicates. Algal cell types were classified using morphological characteristics from previously published microscopy pictures^81–83^. Algae morphology classification is available in Supplementary Fig. 3a, showing all described classes and descriptions.

Across all samples, biovolume was calculated after Hillebrand et al.^84^, assuming spherical (Spherical and Rosette classes) and prolate spheroidal (Oval, Spheroid, Papilla classes) geometries for snow algae cysts. Filamentous Ancylonema, Unicellular Ancylonema, and Trebouxiophyceae-like cells were assumed to have cylindrical geometries. Biovolume calculations for the different cell types combined with FlowCam geodesic length values and additional cell width and length measurements undertaken with ImageJ v 1.53t^85^. For morphologies classified as snow algae, unicellular Ancylonema, and single-cell associated with filamentous Ancylonema, calculations in the snow sample were based on individual cell length and width measurements conducted using ImageJ. Biovolume calculations for the Trebouxiophyceae-like category were based on FlowCam Geodesic length values and width measurements acquired using ImageJ. For algae in the Filamentous Ancylonema class quantified in the ice samples, ~500 length and 100 width measurements of single cells in filaments were conducted in ImageJ and averaged across triplicates for each sample. These averaged width and length ImageJ values were also used to calculate average single-cell biovolumes. The quotient of average single-cell length and the Geodesic length of individual filaments provided the estimated number of Ancylonema cells associated with each filament. For each sample, the total biovolume contributed by algae of the Filamentous Ancylonema was then calculated by multiplying the estimated number of single cells in filaments by the average single-cell biovolume.

### Metabolomics extraction, identification, and normalization

The 19 red snow samples and 9 dark ice samples from the replicate filtrations of the collected five red snow and three dark ice samples were vacuum concentrated (RT, 1200 rpm, 8 mbar, RVC 2–25 CDplus, Christ) to remove methanol, freeze-dried (Scanvac CoolSafe, Labogene), and stored at −80 °C until extraction. Metabolites were extracted with a biphasic extraction protocol and analyzed by gas chromatography-mass spectrometry (GC-MS, MeOH-water extract) and liquid chromatography-mass spectrometry (LC-MS, MTBE extract), respectively. The methods for both metabolite extraction and analysis are available in Peter et al.^19^.

Raw data was pre-processed in MS-DIAL, and annotated LC-MS and GC-MS feature tables were imported into an R workspace (R Core Team 2021). To normalize variance in feature intensity based on technical factors, median peak intensity per sample, extracted sample dry weight, and run order in measurement batch were removed for each metabolic feature using an ANOVA-based approach^86^. We decided on a rank-based comparison of metabolite intensities between red snow and dark ice samples to account for different matrix effects of both sample types on ionization and detection in GC-MS and LC-MS analysis. Normalized intensities were therefore converted to percentages of the metabolome analyzed by GC-MS and LC-MS for each sample.

### Statistical analysis

All plots and statistical analysis were undertaken in R with rstatix v0.7.2^87^, ggplot2 v3.4.2^88^, and ggVennDiagram v1.2.3^89^. Figure panels were assembled and annotated in Inkscape v. 1.3.2.

## Supplementary information

supplementary_information

supplementary_data_1

supplementary_data_2

supplementary_data_3

supplementary_data_4

## Data Availability

The mass spectrometry proteomics data have been deposited to the ProteomeXchange Consortium (http://proteomecentral.proteomexchange.org) via the PRIDE partner repository with the dataset identifier PXD057047. The amplicon sequencing data, the FlowCam cell counts and biovolume measurement datasets, the raw and normalized metabolite datasets, and the protein database are deposited with the GFZ data services (https://dataservices.gfz-potsdam.de/portal/) with the following 10.5880/GFZ.3.5.2024.003.
