# Supplementary material for: Algae-dominated metaproteomes uncover cellular adaptations to life on the Greenland Ice Sheet
Source: NPJ Biofilms Microbiomes. 2025 Sep 9;11:181. doi: 10.1038/s41522-025-00770-2 (PMC12420790; doi:10.1038/s41522-025-00770-2)
Supplement: Supplementary file 1 — supplementary_information [file 41522_2025_770_MOESM1_ESM.pdf]

Supplementary information for:

Algae-dominated metaproteomes uncover cellular adaptations to life on the Greenland Ice Sheet

Helen K. Feord[1]*, Anke Trautwein-Schult[2], Christoph Keuschnig[1], Anne Ostrzinski[2], Elisa K. Peter[1,3], Carsten Jaeger[4], Jan Lisec[4], Rey Mourot[1,3,5], Ravi Sven Peters[1], Ozan Çiftçi[1], Martyn Tranter[6], Alexandre M. Anesio[6], Dörte Becher[2], Liane G. Benning[1,3]*

1. GFZ Helmholtz Centre for Geosciences, 14473 Potsdam, Germany

2. Institute of Microbiology, University of Greifswald, 17489 Greifswald, Germany

3. Department of Earth Sciences, Freie Universität Berlin, 12249 Berlin, Germany

4. Bundesanstalt für Materialforschung und -prüfung, 12489 Berlin, Germany

5. Aix Marseille Univ, Université de Toulon, CNRS, IRD, MIO, Marseille, France

6. Department of Environmental Science, Aarhus University, 4000 Roskilde, Denmark

*Corresponding authors: helen.feord@gfz.de and benning@gfz.de

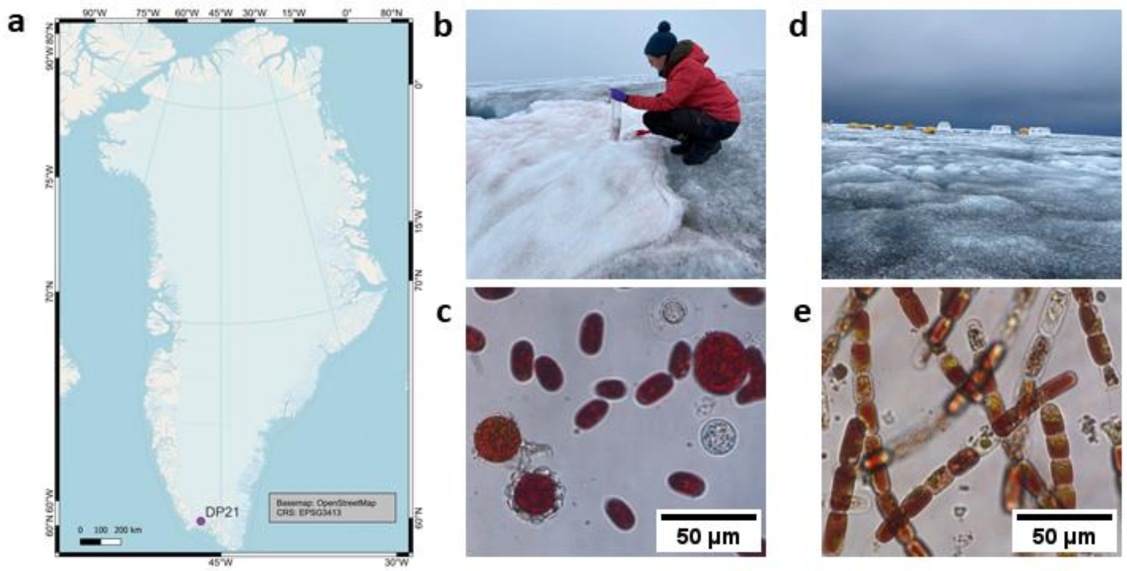

Supplementary Figure 1 Sampling location and sample type. a. Sampling location for both sample types at the DEEP PURPLE ERC ice camp in 2021 61°05’ N,46°50’ W (labelled DP21 on the map, image credit Shunan Feng). **b.** Snow patch sampled for red snow (with person for scale; image credit: Tim Kalvelage), **c.** Microscopy picture representative of red snow chlorophyte algae from the 2021 Greenland DEEP PURPLE ERC field campaign (image credit: Lou-Anne Chevrollier). **d.** Image representative of dark ice substrate similar to the one sampled for the samples used in this manuscript (with tents for scale; image credit: Liane G. Benning). **e.** Microscopy picture representative of dark ice streptophyte algae from the 2021 Greenland DEEP PURPLE ERC field campaign (image credit: Lou-Anne Chevrollier).

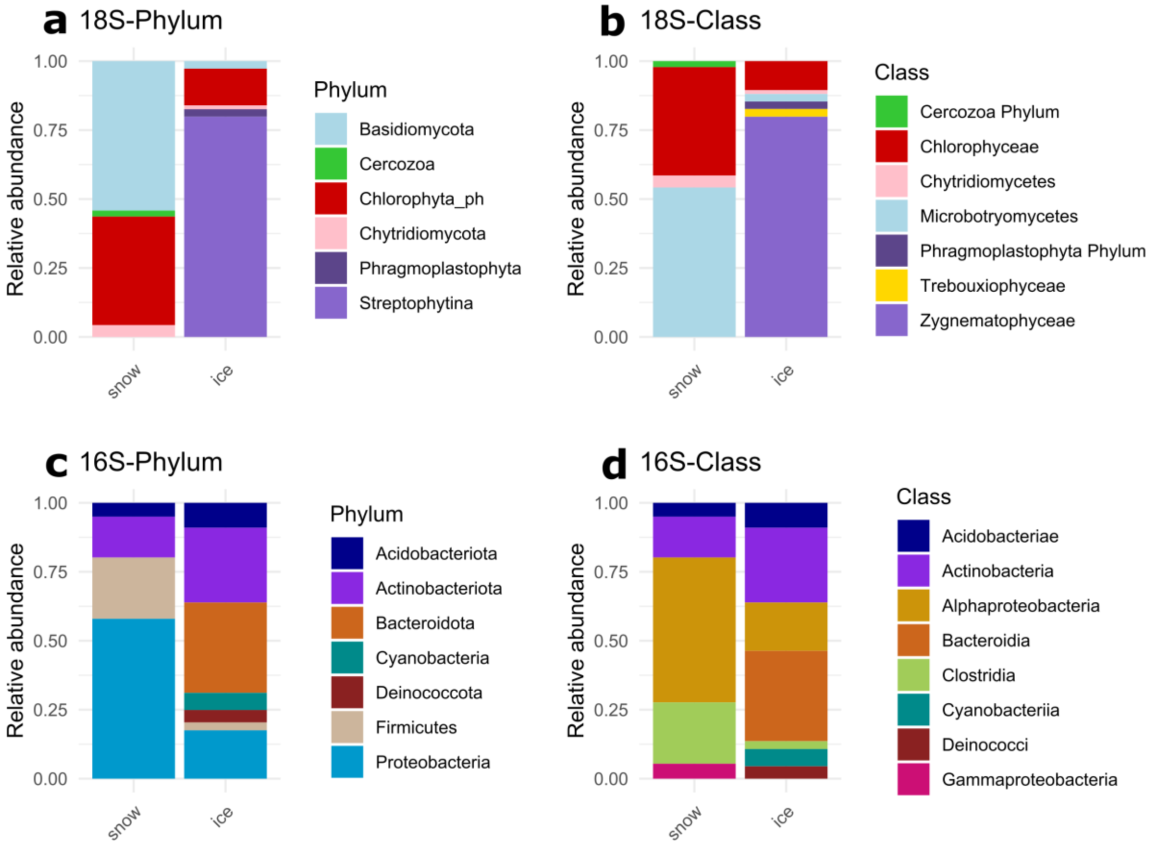

Supplementary Figure 2 Amplicon sequencing of ice and snow samples. Relative abundance of 18S amplicon sequencing at **a.** phylum and **b.** class level, and relative abundance of 16S amplicon sequencing at **c.** phylum and **d.** class level. All ASVs representing under 1% of the sample have been removed for plotting.

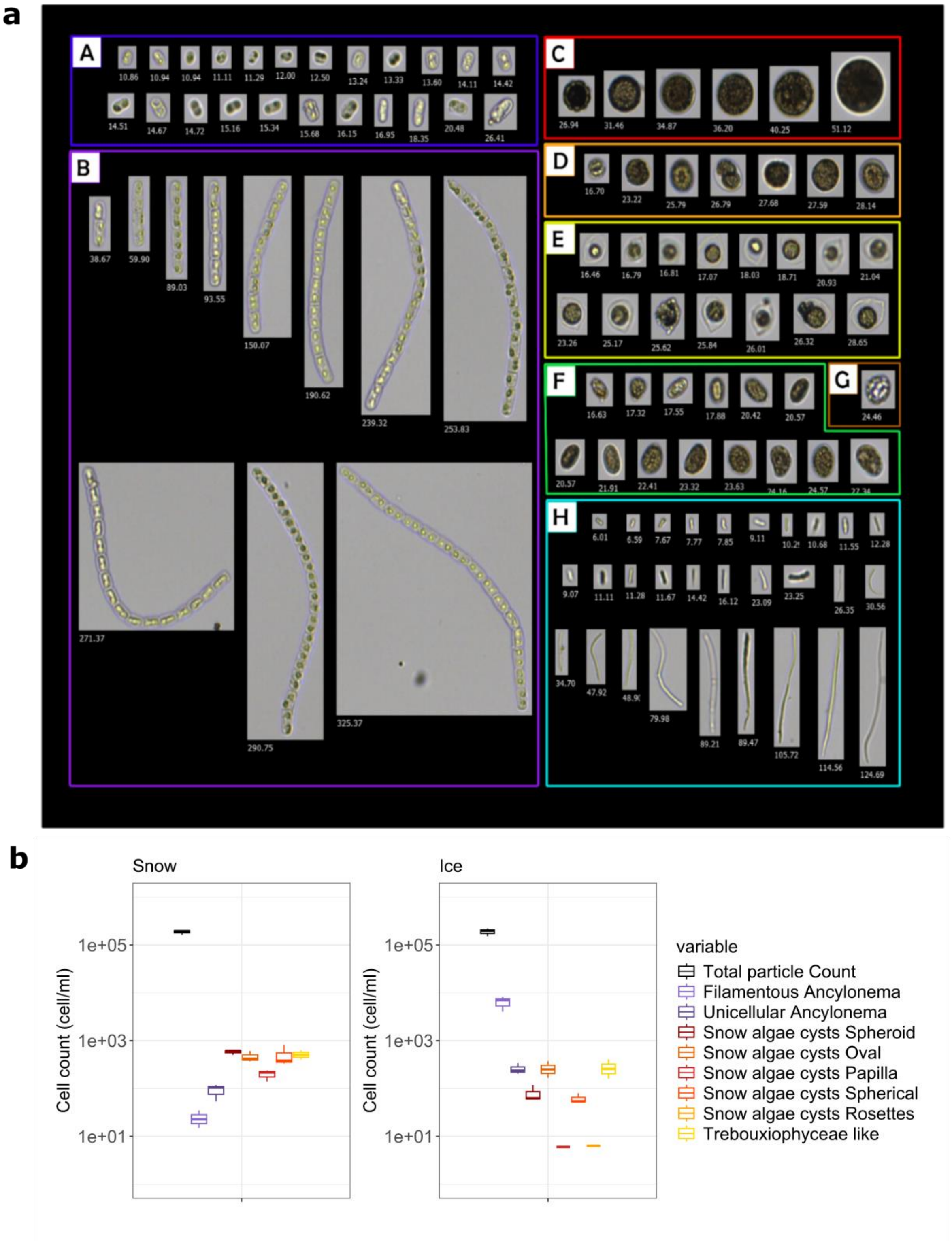

Supplementary Figure 3 Morphology description and cell counts for different categories of algal cell morphologies plotted in Figure 1f. Microscopy and imaging was undertaken with a FlowCam (see online methods). **a.** The different morphologies presented are: A. Unicellular Ancylonema, B. Filamentous Ancylonema, C. Snow algae cysts Spherical, D. Snow algae cysts Spheroid, E. Snow algae cysts Papilla, F. Snow algae cysts Oval, G. Snow algae cysts Rosette, H. Trebouxiophyceae-like. **b.** Cell counts for each morphology in snow and ice samples in cells per ml.

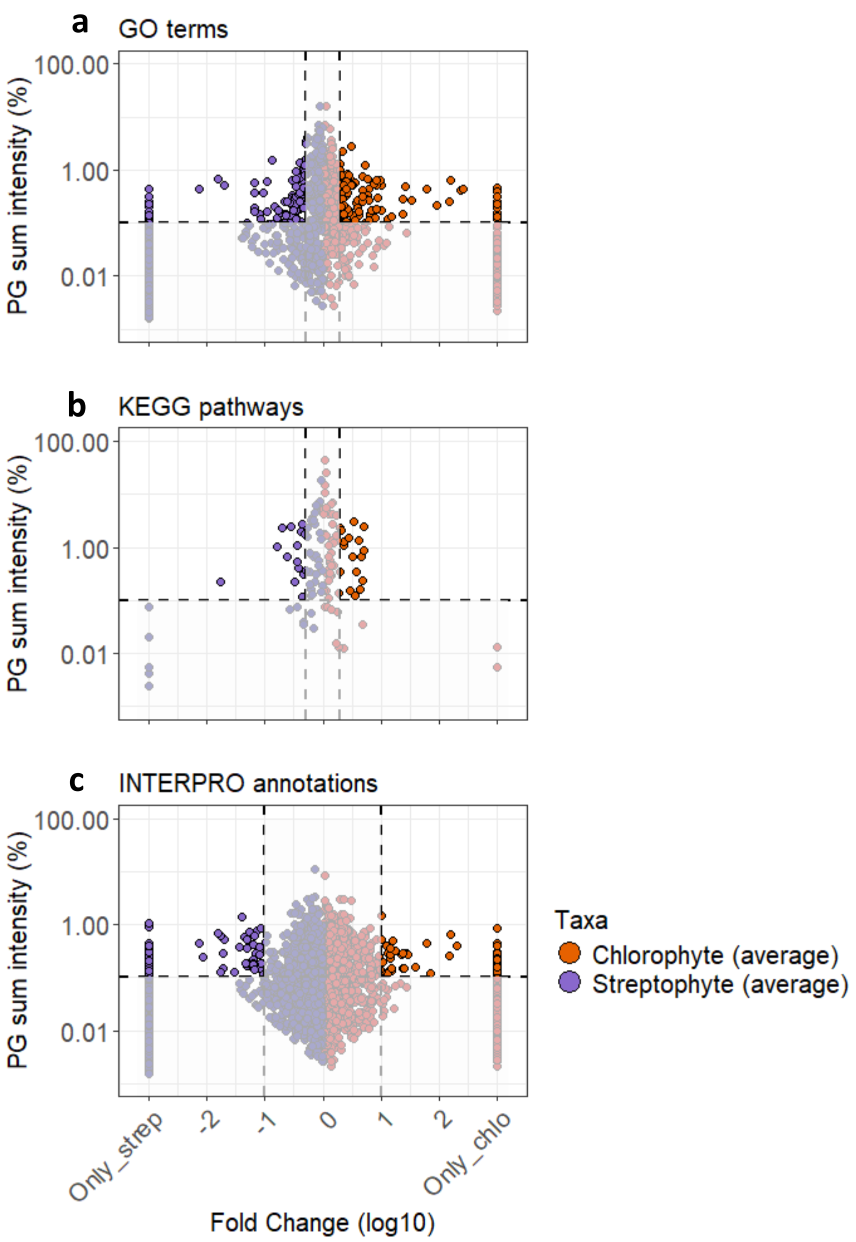

Supplementary Figure 4 Quantity and log10 fold change between the chlorophyte and streptophyte proteome for different PG annotations. Protein annotations with **a.** GO terms, **b.** KEGG pathways, and **c.** INTERPRO annotations plotted according to the log10 fold change between proteome average (x axis) and the PG sum intensity in percentage for the proteome with the highest percentage for each annotation (y axis). Annotations higher in the chlorophyte proteome are plotted in orange and have a positive fold change and annotations higher in the streptophyte proteome are plotted in purple and have a negative fold change. Certain annotations only exist in one proteome and are marked as only chlorophyte ("Only_chlo") or only streptophyte ("Only_strep). Dotted lines indicate the cut-offs used in this study: GO and KEGG pathways are enriched if the log10 fold change ≥ 0.3, sum percentage PG intensity in the enriched proteome ≥ 0.1%, and INTERPRO annotations are enriched in a proteome if the log10 fold change ≥ 1 and the sum percentage PG intensity in the enriched proteome ≥ 0.1%. Abbreviation: PG = Protein group.

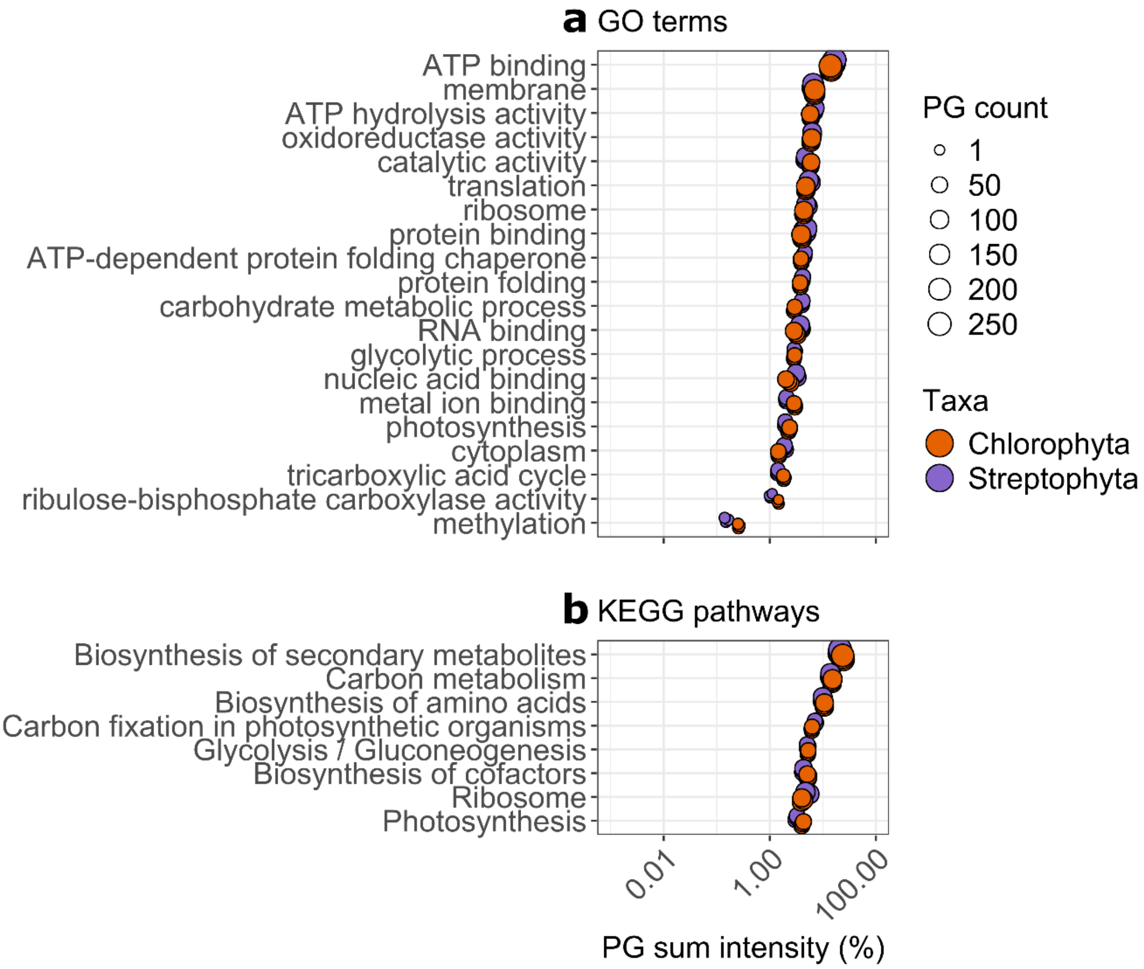

Supplementary Figure 5 Select PG GO terms and KEGG pathways that do not differ between sample type. **a.** 20 select GO terms and **b.** 8 select KEGG pathways with no significant enrichment in either proteomes (log10 FC < 0.3).The full set of KEGG and GO terms is provided in Supplementary information 2 and 3. Abbreviation: PG = Protein group.

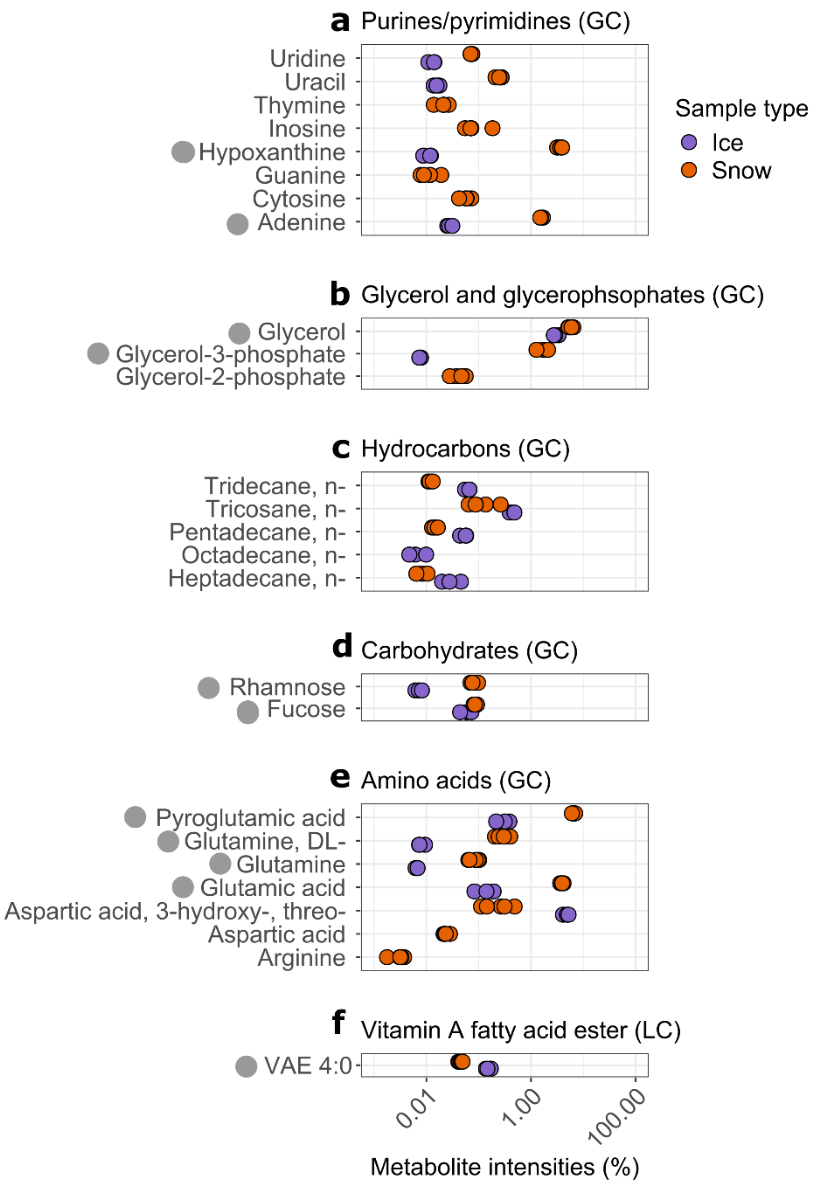

Supplementary Figure 6 Normalised metabolite intensities in snow (n = 5) and ice (n=3) samples. Metabolites are plotted according to metabolite intensity (normalized for sample weight, median peak intensity, and run order using ANOVA based factor removal, grouped by sample type), plotted as the percentage (%) of total metabolites for each sample in either GC/MS or LC/MS acquired data. Presented data includes **a.** Purines/pyrimidines (GC), **b.** Glycerol and glycerophosphates (GC), **c.** Hydrocarbons (GC), **d.** Carbohydrate (GC), **e.** Amino acids (GC), and **e.** Vitamin A fatty acid ester (LC). Grey circles indicate metabolites that are referred to in the main text. GC,Gas Chromatography; LC,Liquid Chromatography.

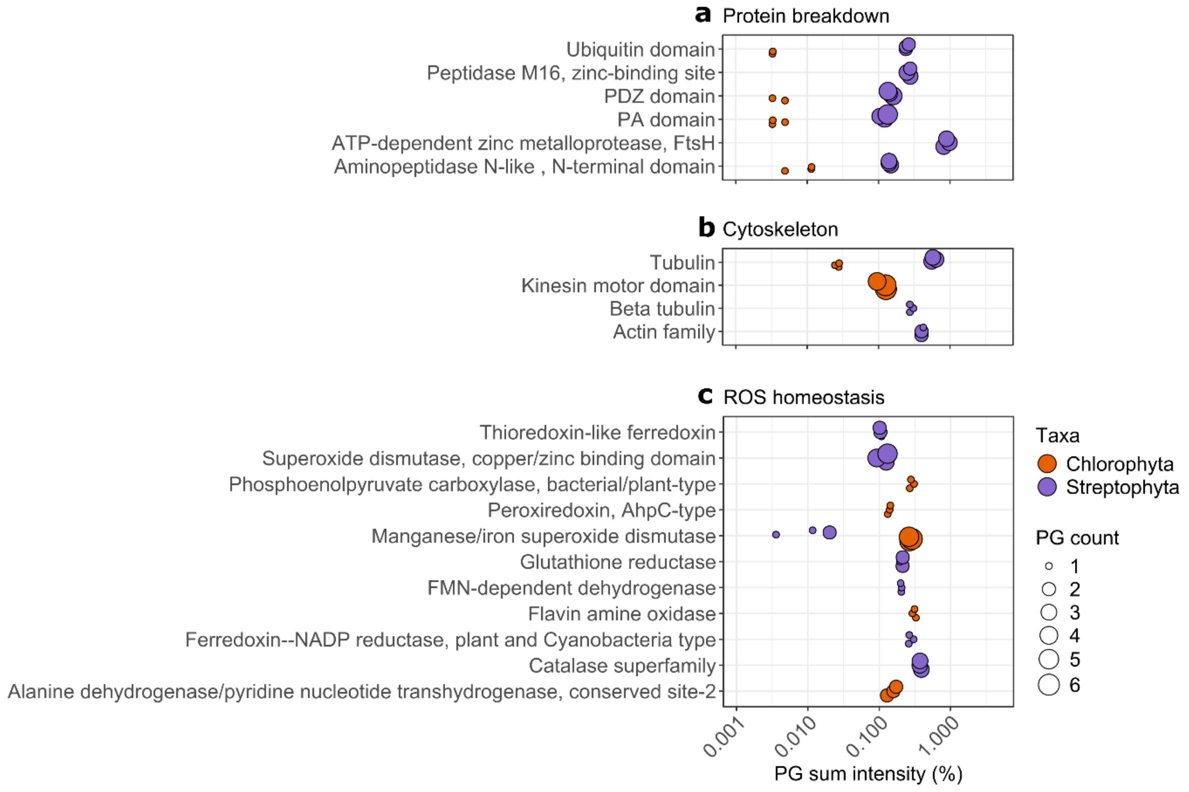

Supplementary Figure *7* Protein groups functions with siginificantly different abundance in the chlorophyte vs streptophyte proteome. Protein groups associated with **a.** protein breakdown, **b.** the cytoskeleton, **c.** Methylation, and **d.** ROS homeostasis. Functional groups are expressed as the percentage of proteins with such an annotation in each proteome. Function was assigned with INTERPRO domains. The number of proteins associated with each function (PG count) represented with the size of the symbol. All protein functions shown are significantly enriched in one proteome ($p < 0.05$, Log10 FC > 1, PG sum intensity > 0.1% in the sample enriched in the annotation). Abbreviations: PG, Protein group; ROS, Reactive Oxygen Species; FC, Fold Change

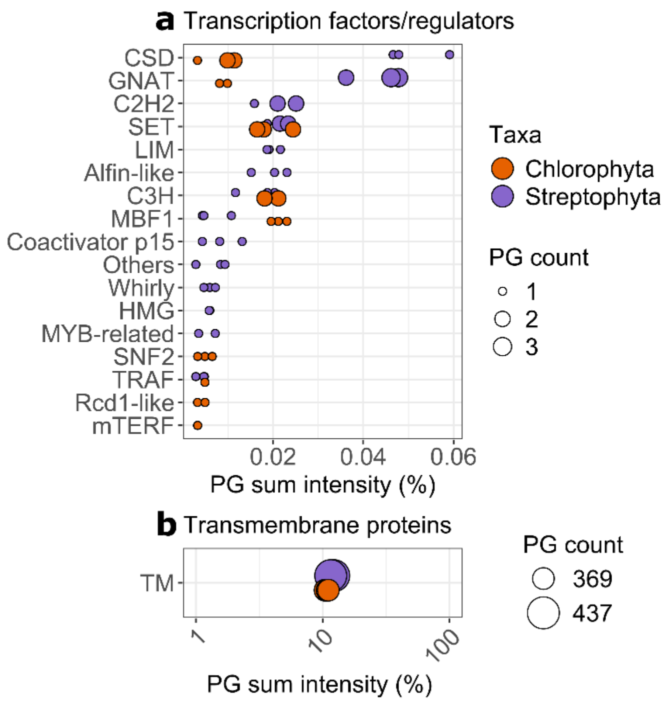

Supplementary Figure 8 PGs identified as transcription factors/regulators and transmembrane proteins. a. Transcription factors/regulator PGs (identified with iTAK; see online methods), and **b.** Transmembrane PGs (identified with TMHMM; see online methods). Functional groups are expressed as the percentage of proteins with such an annotation in each proteome. The number of proteins associated with each function (PG count) represented with the size of the symbol. Abbreviation: PG, Protein group.

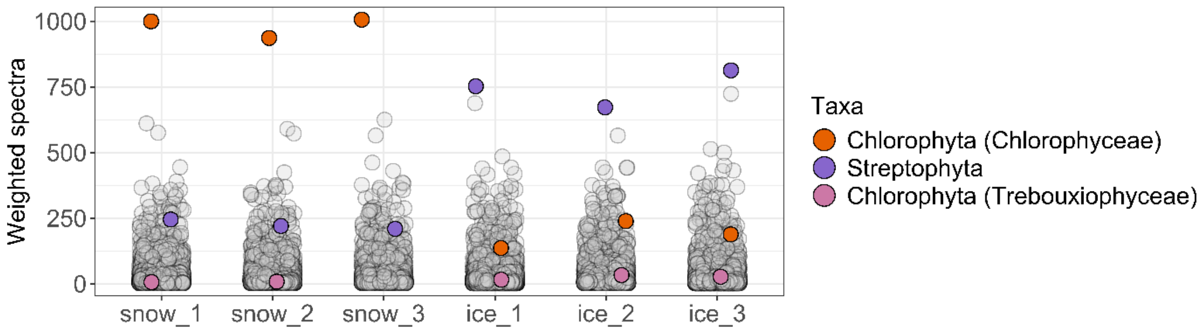

Supplementary Figure 9 Abundance of rbcL PGs in the total metaproteomes of the 3 snow and 3 ice samples quantified in this study. All PGs quantified in each sample are plotted, all points represent 1 PG (including non-algae PGs), plotted with weighted spectra abundance. Orange points are the rbcL PGs identified as Chlorophyta (Chlorophyceae), purple as Streptophyta, and pink Chlorophyta (Trebouxiophyceae) with MEGAN. All other PGs are grey. Abbreviations: PG,Protein group.

Supplementary Table 1 Taxonomy of databases sequences used in this study, as assigned by BLASTp against NCBI nr and MEGAN (see online methods). "Not assigned" refers to sequences with no assigned taxonomy, "NCBI" refers to sequences that could not be distinguished between viruses and cellular organisms, and "Cellular organism" refers to sequences that could not be assigned beyond cellular organism. Approximately half the database sequences were aligned with BLASTp.

Taxonomy assigned with MEGAN	Number of sequences	Category in Figure 1
Total database sequences	437423	
Sequences aligned with BLASTp	225078	
Chlorophyta	58910	Chlorophyta
Streptophyta	49137	Streptophyta
Fungi	43800	Fungi
Eukaryota	17581	Other
Bacteria	14547	Prokaryota
Viridiplantae	13675	Viridiplantae other
cellular organisms	10255	Other
Sar	6216	SAR
Not assigned	3324	Other
Metazoa	3109	Metazoa
Opisthokonta	1892	Other
NCBI	838	Other
Amoebozoa	810	Amoebozoa
Archaea	338	Prokaryota
Viruses	315	Virus
Discoba	167	Other
Apusozoa	44	Other
Haptista	43	Other
Rhodophyta	30	Other
Choanoflagellata	25	Other
Filasterea	22	Other

Supplementary note 1: Further discussion on protein groups linked to photosynthesis

Both the studied algae taxa (snow algae and glacier ice algae) actively fix carbon while thriving on the GrIS. However, the high abundance of rbcS in the streptophyte proteome (**Fig. 3d**) could have an influence on the catalytic activity of RuBisCO as a whole[1], indicating that the streptophyte algae may be photosynthetically more active at the time of sampling. This is consistent with previous reports of lower chlorophyll concentrations and photosynthetic performances in the snow algae compared to glacier ice algae[2,3]. The snow algae, in hypnozygote stage, are not actively dividing, and thus likely require less organic carbon compared to the streptophyte algae in vegetative stage. The high expression of carbonic anhydrases points towards dissolved inorganic carbon limitation in both snow and ice habitats (**Fig. 3d**), concurring with previous findings for snow algae[4,5]. Both algal groups could also use a substantial amount of their fixed carbon to produce extracellular polysaccharides, as evidenced by the two high abundance PGs linked to fucose and rhamnose production, in the streptophyte and chlorophyte proteomes, respectively[6].

References:

1. Mao, Y. *et al.* The small subunit of Rubisco and its potential as an engineering target. *J. Exp. Bot.* **74**, 543–561 (2023).

2. Lutz, S., Anesio, A. M., Edwards, A. & Benning, L. G. Linking microbial diversity and functionality of arctic glacial surface habitats. *Environ. Microbiol.* **19**, 551–565 (2017).

3. Halbach, L. *et al.* Pigment signatures of algal communities and their implications for glacier surface darkening. *Sci. Rep.* **12**, 17643 (2022).

4. Ezzedine, J. A. *et al.* Adaptive traits of cysts of the snow alga Sanguina nivaloides unveiled by 3D subcellular imaging. *Nat. Commun.* **14**, 7500 (2023).

5. Hamilton, T. L. & Havig, J. R. Addition of dissolved inorganic carbon stimulates snow algae primary productivity on glacially eroded carbonate bedrock in the Medicine Bow Mountains, WY, USA. *FEMS Microbiol. Ecol.* **99**, fiad056 (2023).

6. Xiao, R. & Zheng, Y. Overview of microalgal extracellular polymeric substances (EPS) and their applications. *Biotechnol. Adv.* **34**, 1225–1244 (2016).
